# Machine Learning Model Integrating Computed Tomography Image–Derived Radiomics and Circulating miRNAs to Predict Residual Teratoma in Metastatic Nonseminoma Testicular Cancer

**DOI:** 10.1200/CCI-25-00105

**Published:** 2025-08-25

**Authors:** Guliz Ozgun, Neda Abdalvand, Gizem Ozcan, Ka Mun Nip, Nastaran Khazamipour, Arman Rahmim, Robert Bell, Corinne MauriceDror, Maryam Soleimani, Kim Chi, Bernhard J. Eigl, Craig Nichols, Christian Kollmannsberger, Ren Yuan, Lucia Nappi

**Affiliations:** ^1^ BC Cancer Vancouver Center, Department of Medical Oncology, Vancouver, BC, Canada; ^2^ BC Cancer Research Center, Vancouver, BC, Canada; ^3^ Vancouver Prostate Centre, Vancouver, BC, Canada; ^4^ Testicular Cancer Commons, Beaverton, OR; ^5^ BC Cancer Vancouver Center, Department of Radiology, Vancouver, BC, Canada

## Abstract

**PURPOSE:**

Chemotherapy is the primary treatment for metastatic nonseminomatous germ cell tumors (mNSGCTs), but patients often encounter postchemotherapy residual disease. Accurate noninvasive methods are needed to predict the histology of these masses, guiding treatment and reserving surgery for those with teratoma. This study aims to enhance predictive accuracy by integrating computed tomography (CT) radiomics features with miRNAs (miR371-375) to distinguish between teratoma and nonteratoma histology in postchemotherapy residual masses.

**METHODS:**

We retrospectively identified 111 lesions, divided into training and test sets (n = 78 *v* 33) with equal class distribution. 3D Slicer was used to segment lesions with a short axis of >10 mm from the postchemo-presurgical CT images, and radiomics features were extracted. Presurgery plasma miR371-375 levels were measured by real-time polymerase chain reaction. Four machine learning models evaluated the predictive value of radiomics alone (R-only) and combined with miR371-375 levels, and the best performer was selected. Clinical factors associated with teratoma from univariate analysis were included in multivariate analysis with the best radiomics signature to assess their impact on predicting teratoma histology.

**RESULTS:**

The CatBoost (CB) model R + 371 + 375 exhibited the best and most robust overall accuracy for predicting residual teratoma, with the highest AUC values (0.96, 95% CI, 0.88 to 1.0 for training, 0.83, 95% CI, 0.68 to 0.98 for testing) and a well-balanced sensitivity and specificity. Univariate analysis identified presurgery alpha-fetoprotein (*P* = .01), beta-human chorionic gonadotropin (*P* = .01), initial teratoma pathology (*P* = .01), and lymph node metastases (*P* = .02) as significant predictors for teratoma. Multivariate analysis included these features and the radiomics signature, which was the strongest independent predictor (*P* < .0001).

**CONCLUSION:**

Combining miR371-375 with CT radiomics features improves the accuracy of predicting teratoma histology of postchemotherapy residual disease in mNSGCTs and, therefore, has the potential to guide treatment decision making.

## INTRODUCTION

Testicular cancer is classified into two main histological subtypes, seminoma and nonseminoma (including embryonal carcinoma, yolk sac tumor, choriocarcinoma, and teratoma, mixed together or sometimes with seminoma histology), and is the most common solid tumor in young men age 15-45 years.^1-3^ Testicular cancer also shows molecular heterogeneity among the various histology subtypes: embryonal carcinomas often overexpress pluripotency-related gene while teratomas express genes related to somatic differentiation.^4,5^ As an example, AGR2 and KRT19 proteins and their related genes were recently shown to be differently expressed between teratoma and benign tissue in the postchemotherapy resection material.^6^ Fortunately, regardless of the subtype, testicular cancer can be effectively treated with chemotherapy, often in conjunction with surgery or radiation therapy. Patients with mNSGCT often have residual disease after chemotherapy, and resection of any residual mass measuring over 10 mm is recommended because of the risk of growing teratoma syndrome and malignant somatic transformation of residual teratomas, both characterized by poor response to chemotherapy and limited treatment options.^7-9^ However, the current treatment approaches to address the possible residual teratoma can lead to overtreatment and thus unnecessary complications, as benign lesions such as fibrosis are identified in about 50% of patients, while teratoma and viable germ cell tumors (vGCTs) are identified in 40% and 15% of patients, respectively.^10,11^ Therefore, accurate histology prediction models are needed to guide patient selection for different treatment strategies.

**CONTEXT**

**Key Objective**
Can the integration of radiomics and microRNAs data enhance the accuracy of histological prediction of postchemotherapy residual disease in metastatic nonseminomatous germ cell tumors (mNSGCTs)?
**Knowledge Generated**
This study demonstrates that integrating computed tomography radiomics features with circulating miR371 and miR375 provides a powerful approach to distinguish teratoma from nonteratoma histology in mNSGCT patients with postchemotherapy residual disease. The combined model achieved strong predictive performance, outperforming clinical parameters alone, and represents a promising step toward more personalized and precise treatment planning in testicular cancer care.
**Relevance *(J. Warner)***
This predictive model built on readily acquired clinical data provides encouraging results for the detection of residual disease. External validation and prospective studies implementing the model in a clinical decision-making pathway should be considered next.*
*Relevance section written by *JCO CCI* Editor-in-Chief Jeremy Warner, MD, MS, FAMIA, FASCO.

Computed tomography (CT) and magnetic resonance imaging cannot predict the histology of the residual disease after chemotherapy. Fluorodeoxyglucose (FDG)-positron emission tomography-CT offers limited utility in mNSGCTs, especially when the lesions have intermediate FDG uptake.^12,13^ Biopsy of the residual lesions is invasive, could have sample bias, and is not always feasible. To address these limitations, ongoing efforts aim to integrate artificial intelligence (AI) for pattern recognition and quantitative analysis of medical images with biomarkers to improve diagnostic accuracy.

Radiomics is a developing field of quantitative imaging, converting medical images to mineable data.^14-16^ So far, radiomics has been shown to be a valuable tool for predicting histology, treatment response, and prognosis.^17-21^ In mNSGCTs, radiomics presented promising results in predicting malignant retroperitoneal lymph nodes (RPLN).^22-24^ These studies have primarily focused on the radiomics-only approach to discriminate malignant (vGCT + teratoma) versus benign lesions. However, the most relevant and still unmet clinical need is determining the teratoma component that requires surgery to be eradicated.^8^

Studies on novel circulating biomarkers, such as miRNAs 371-373 and 302/367 clusters, have been conducted in testicular GCTs.^25^ Among these, miRNA-371a-3p (miR371) has shown the highest accuracy in detecting vGCTs.^26,27^ Thus, large prospective clinical trials, such as the SWOG S1823 and the COG 1531, were set up, aiming to validate miR371 to predict tumor relapse in patients with early-stage GCTs.^28,29^ Despite encouraging results, miR371 is unable to detect teratoma. Another microRNA, miR375, has shown higher expression in teratoma than in other vGCTs. Combining miR371 and miR375 to differentiate GCT histologic subtypes has improved accuracy, yet it is suboptimal to be proposed as a clinical tool.^30^

Recently, given the limited efficacy of single-omics data, there has been a growing interest in combining different approaches to enhance accuracy.^31^ Within this context, integrating radiomics with biomarkers such as miRNAs to predict teratoma histology remains underexplored, necessitating further studies to establish clinical utility. Built on our previous work^30^ and the potentially promising role of radiomics in medical imaging, we hypothesize that integrating CT-driven radiomics features with miR371 and miR375 may enhance the predictive accuracy in identifying teratoma in mNSGCT patients with postchemotherapy residual masses to accurately select patients for surgery.

## METHODS

### Patient Selection Criteria

**FIG 1.**
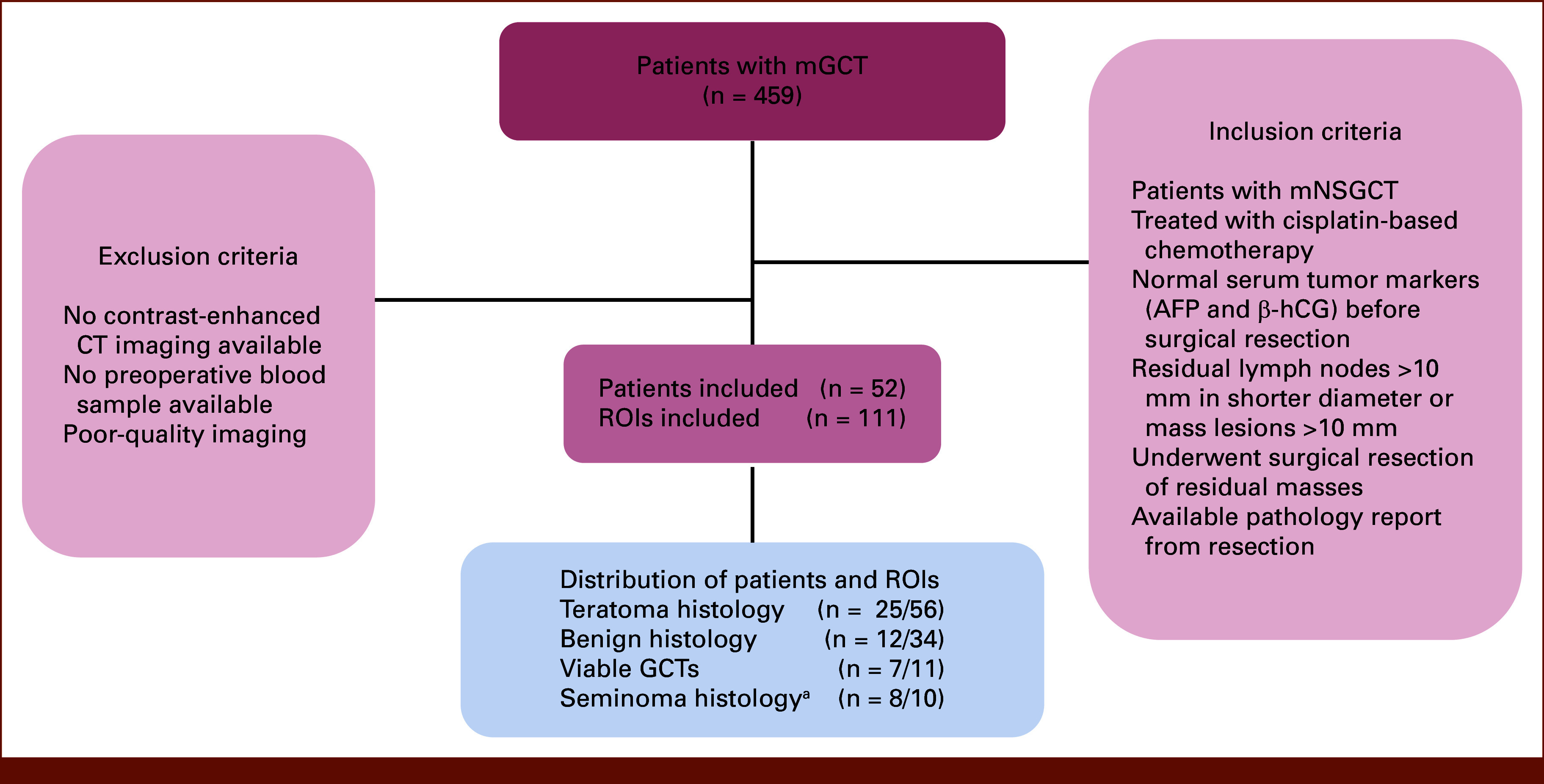
The flow diagram illustrating the participants enrolled in the study. ^a^Seminoma cases with available pre-surgery blood samples were included for AI training purposes. AFP, alpha-fetoprotein; β-hCG, beta-human chorionic gonadotropin; CT, computed tomography; GCT, germ cell tumor; mGCT, metastatic germ cell tumor; mNSGCT, metastatic nonseminomatous GCT; ROI, region of interest.

The study was approved by the University of British Columbia Ethical Board (H18-01484). A retrospective data set from the British Columbia Cancer GU biobank was compiled that satisfied the following eligibility criteria: males who were diagnosed with mNSGCT and had residual disease larger than 10 mm (ie, residual masses over 10 mm in diameter and lymph nodes over 10 mm short axis) after cisplatin-based chemotherapy, with normal serum tumor markers before surgical resection (alpha-fetoprotein [AFP]: <8.4 µg/L and beta-human chorionic gonadotropin (β-hCG): <3 IU/L) and underwent surgical resection of residual disease, with available presurgical blood samples and CT scan and pathology reports of the resected lesions. Patients were excluded if their presurgical CT did not have intravenous contrast, had poor imaging quality, or the residual masses had not been adequately assessed (Fig 1). Seminoma cases with available presurgery blood samples were included for AI training purposes.

### Radiomics Signature Design

Residual lesions were identified on the CT images by two independent investigators (R.Y. and G.O.) who also reviewed the CT reports and correlated the CT images with the surgical pathology. After this extensive review, the residual lesions were labeled as teratoma or nonteratoma.

3D Slicer (version 5.6.1)^32^ was used to manually segment regions of interest (ROIs) corresponding to residual masses with diameter ≥10 mm (or short axis ≥10 mm in lymph nodes; Data Supplement, Fig S1). Radiomics features were extracted using the Radiomics extension in 3D Slicer, which is based on the PyRadiomics library.

**FIG 2.**
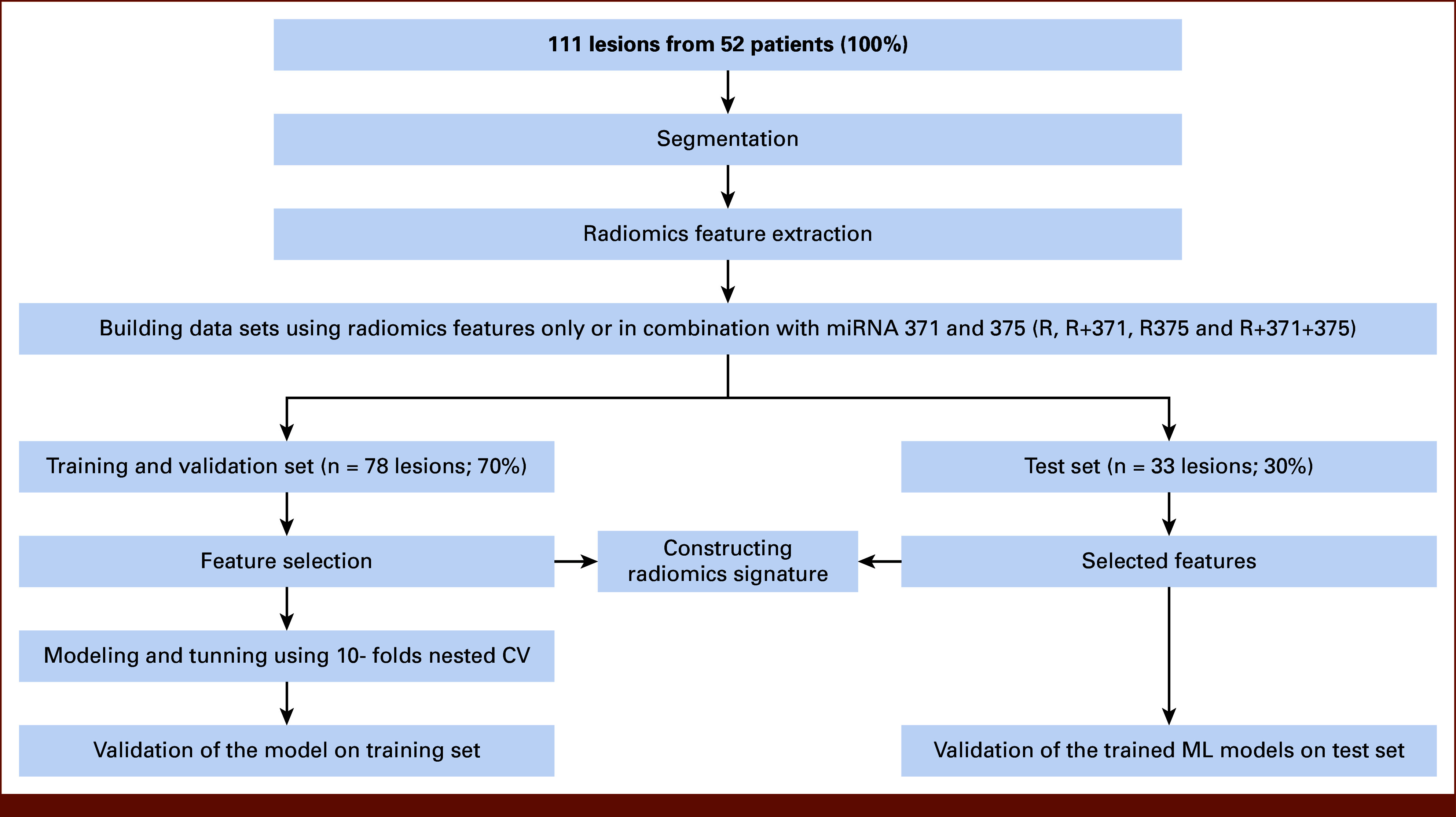
Schematic diagram of the study roadmap. The workflow starts with image acquisition, segmentation, and feature extraction. Features are selected, and ML models are developed. The most effective model is determined through evaluation, while radiomics signatures are calculated separately. CV, cross-validation; ML, machine learning.

We used LassoCV for feature selection, shrinking less significant features' coefficients to zero. After identifying the most relevant radiomics features and defining radiomics signatures, machine learning (ML) models were developed and subjected to comprehensive evaluation to determine the best-performing model for accurate teratoma histology prediction (Fig 2).

Radiomics (R) features were integrated with miR371-375 (371 and 375) in different combinations, creating four separate data sets: R, R + 371, R + 375, and R + 371 + 375.

### Classifier Construction

Four commonly used feature selection algorithms were used to identify discriminating features: CatBoost (CB), Random Forest (RF), Gradient Boosting (GB), and Support Vector Machine (SVM). Each has distinct strengths with the specific data set construction in this study.^33-36^ After the evaluation of four ML models, the best ML model, CB was selected because of its superior performance. We implemented nested cross-validation (CV) using stratified 10-fold CV to achieve robust model evaluation.

### miRNA Analysis: RNA Extraction and Real-Time Polymerase Chain Reaction Analysis

The miRNA analysis was conducted in line with the methodology established in our previous works.^30,37^ Blood samples were obtained from the British Columbia Cancer GU biobank under standardized collection and processing protocols within 8-12 weeks of treatment initiation.

Detailed methodology is included in the Data Supplement.

### Statistical Analysis

All data were entered into an Excel spreadsheet securely stored on our local, password-protected BC Cancer server. The data were deidentified before conducting statistical analysis. Descriptive statistics was used to describe the patient characteristics.

Seventy-eight samples (39 teratomas and 39 nonteratomas) were allocated to the training set, and 33 (17 teratomas and 16 nonteratomas) were added to the test set, maintaining equal class distribution in both sets. The training set was used for feature selection and model development, while the test set remained completely unseen during training.

Model performances were evaluated on the training and unseen test sets using receiver operating characteristic (ROC) curves, with the AUC as the primary performance metric. Confidence intervals for the AUC values are computed through bootstrap sampling with 1,000 iterations, enabling the assessment of variability in model performance. Additionally, sensitivity (SN), specificity (SP), positive predictive value (PPV), and negative predictive value (NPV) are reported.

To assess whether the sample mean significantly differs from the population mean, a Z-test was conducted (Data Supplement, Table S2). The model with the highest AUC was identified to determine the best-performing approach.

Univariate analysis was conducted to investigate the predictive value of clinical factors (ie, teratoma histology in the primary, prechemotherapy tumor markers—AFP, β-hCG, and lactate dehydrogenase–, lesion size, and lesion location).^38^ Finally, a multivariate analysis was used to determine the independent impact on teratoma prediction. Statistical significance was defined as two-tailed *P* < .05 for all tests.

This study was approved by the University of British Columbia Ethical Board (H18-01484). Informed consent from each participant or each participant's guardian was obtained before enrolling the patients in the research study.

## RESULTS

### Patients and Sample Characteristics

On the basis of the inclusion criteria, 52 patients were identified, with a median age of 33 years (IQR, 23-66). These patients contributed a total of 111 lesions, including teratoma (n = 56), fibrosis/necrosis (n = 34), vGCT (n = 11), and seminoma lesions (n = 10). The lesions were distributed across various sites: 87 lymph nodes (n = 68 in the retroperitoneum, n = 11 in the mediastinum, n = 4 in the pelvic region, and n = 4 in the neck), lung (n = 21), and brain (n = 3). The median lesion size was 20 mm (15-32 mm) longitudinally and 27 mm (16-40 mm) transversely, with an interquartile range of 12-27.3 mm (Table 1).

**TABLE 1.** Characterization of Patients and Lesions Included in the Study

Characteristic	Total Population	Necrosis/Fibrosis	Teratoma	vGCT and Seminoma
Patient population, No.	52	12	25	15
Residual masses, No.	111	34	56	21 (11 + 10)
Age at diagnosis, years, median (IQR)	33 (23-66)	32 (27-66)	28 (20-47)	33 (23-56)
Longitudinal size of lesion (diameter, in mm)	20 (15-32)	16 (14-23)	27 (16-40)	18 (15-25)
Transverse size of lesion (diameter, in mm)	27 (16-40)	20 (15-29)	33 (19-45)	24 (20-33)
Presence of histologies in the primary tumor, No. (%)				
Teratoma	51 (45.9)	10 (19.6)	33 (64.76)	8 (15.6)
Seminoma	43 (38.7)	8 (18.6)	23 (53.48)	12 (27.9)
Yolk sac	50 (45)	17 (34)	28 (56)	5 (10)
Embryonal carcinoma	45 (40.5)	11 (24.4)	31 (68.8)	3 (6.66)
Choriocarcinoma	13 (11.7)	7 (53.8)	3 (23.07)	3 (23.07)
Unknown	18 (16.2)	14 (77.77)	2 (11.11)	2 (11.11)
Anatomic locations, No. (%)				
Lymph nodes	87 (78.38)	20(59)	49 (87.5)	18 (85.71)
Retroperitoneum	68 (61.26)	17 (50)	35 (62.5)	16 (76.19)
Pelvis	4 (3.6)	1 (2.94)	3 (5.36)	—
Mediastinum	11 (9.91)	2 (5.88)	8 (14.29)	1 (4.76)
Neck	4 (3.6)	—	3 (5.36)	1 (4.76)
Lung	21 (18.92)	14 (41.18)	7 (12.5)	—
Brain	3 (2.7)	—	—	3 (14.29)

Abbreviation: vGCT, viable germ cell tumor.

### Four Model Performances for Radiomics Feature Selection

Among the ML models, CB showed the highest AUC values on both the training and test sets. The CB performance metrics are presented in Table 2, while the performance metrics for the other ML models are included in the supplementary section (Data Supplement, Tables S1 and S2) with the miRNAs-only model performance (Data Supplement, Tables S3 and S4, and Figs S2 and S3).

**TABLE 2.** The Performance of the CatBoost Model on the Training and Test Sets Across All Data Sets

Performance Metric	R		R + 371		R + 375		R + 371 + 375	
	Training	Test	Training	Test	Training	Test	Training	Test
AUC	0.89 (0.79-0.99)	0.81 (0.65-0.98)	0.91 (0.84-0.97)	0.81 (0.66-0.96)	0.95 (0.87-1.00)	0.82 (0.66-0.97)	**0.96** (0.88-1.00)	**0.83** (0.68-0.98)
Sensitivity	0.89 (0.78-0.99)	0.76 (0.55-0.98)	0.9 (0.76-0.99)	0.82 (0.63-1.0)	0.78 (0.65-0.94)	0.71 (0.53-0.87)	**0.88** (0.74-0.98)	**0.71** (0.57-0.89)
Specificity	0.79 (0.70-0.88)	0.65 (0.42-0.87)	0.52 (0.35-0.79)	0.47 (0.24-0.71)	0.92 (0.84-1.0)	0.65 (0.49-0.84)	**0.93** (0.88-1.0)	**0.76** (0.68-0.93)
NPV	0.86 (0.79-0.95)	0.73 (0.5-0.97)	0.84 (0.71-0.96)	0.73 (0.55-0.87)	0.81 (0.69-0.98)	0.69 (0.53-0.85)	**0.9** (0.78-1.0)	**0.72** (0.61-0.86)
PPV	0.85 (0.80-0.94)	0.68 (0.47-0.89)	0.69 (0.48-0.83)	0.61 (0.49-0.81)	0.92 (0.84-1.0)	0.67 (0.5-0.79)	**0.93** (0.89-1.0)	**0.75** (0.67-0.91)

NOTE. The metrics reported include AUC, sensitivity, specificity, NPV, and PPV, presented as means with 95% CI. Bolded values indicate performance metrics for the R + 371 + 375 combination group in both the training and test sets.

Abbreviations: NPV, negative predictive value; PPV, positive predictive value.

### CB Model on R-Only Data Set

CB exhibited the highest discriminatory ability between teratoma and nonteratoma samples, maintaining consistently superior AUC values on both the training (0.89 [95% CI, 0.79 to 0.99]) and test sets (0.81 [95% CI, 0.65 to 0.98]). Regarding additional performance metrics, again the CB model demonstrated a high SN of 0.89 (95% CI, 0.78 to 0.99) and SP of 0.79 (95% CI, 0.70 to 0.88) on the training set. Moreover, the NPV and PPV were also strong at 0.86 (95% CI, 0.79 to 0.95) and 0.85 (95% CI, 0.80 to 0.94), respectively, indicating robust overall predictive capabilities. However, when evaluated on the test set, all metrics exhibited a mild while expected decline, with SN decreasing to 0.76, SP to 0.65, NPV to 0.73, and PPV to 0.68.

### Combination of R With miR371 and miR375 (R + 371 + 375)

**FIG 3.**
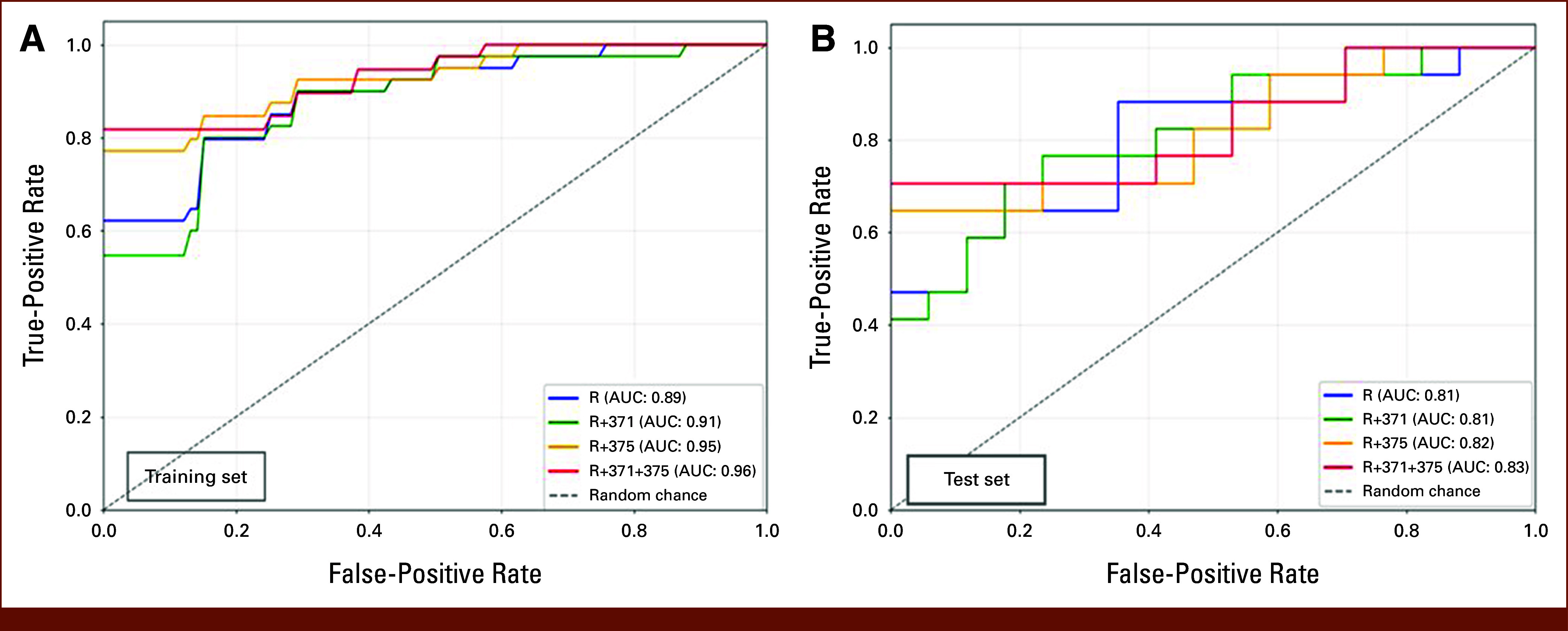
ROC curves of CB radiomics and miRNAs across (A) training and (B) test sets. CB, CatBoost; R, radiomics-only; R + 371, radiomics + miR371; R + 375, radiomics + miR375; R + 371 + 375, radiomics + both miR371 and 375; ROC, receiver operating characteristic.

An improvement in AUC was observed from R (0.89 in training; 0.81 in testing) to R + 371 + 375 (0.96 [95% CI, 0.88 to 1.0], in training; 0.83 [95% CI, 0.68 to 0.98], in testing) for predicting teratoma histology, indicating an enhanced overall discriminative ability. Although SN remained relatively consistent across models, the R + 371 + 375 data set demonstrated superior SP performance (0.93 in training and 0.76 in testing). Both NPV and PPV improved from R to R + 371 + 375, with the latter achieving the highest test PPV of 0.75 (Table 2 and Fig 3).

Overall, despite each model exhibiting distinct strengths and weaknesses, R + 371 + 375 emerged as the most balanced and improved performer. The combined CB model maintained high SN (0.88 in training and 0.71 in testing) while achieving the highest SP (0.93 in training and 0.76 in testing). It also exhibited the best NPV (0.90 in training and 0.72 in testing) and PPV (0.93 in training and 0.75 in testing).

### Clinical Factors and R and microRNAs to Predict Teratoma

Known clinical factors associated with teratoma in the residual disease were tested to predict teratoma in this study. Univariate analysis showed that prechemotherapy AFP levels (odds ratio [OR], 1.0, *P* = .01), β-hCG levels (OR, 0.99, *P* = .01), presence of teratoma in the primary (OR, 2.75, *P* = .01), and metastatic lesion being lymph node (OR, 3.13, *P* = .02) were statistically significant. However, in the multivariate analysis, the combination of R and miRNAs (radiomics signature) was the strongest predictor in both the training and test settings (*P* < .0001 and *P* = .02, respectively; Table 3).

**TABLE 3.** Univariate and Multivariate Analyses of Predictive Factors

Feature	Univariate Analysis (full data set) Logistic Regression *P* value	Multivariate Analysis (training set)	Multivariate Analysis (test set)
Prechemotherapy AFP (µg/L)	OR, 1.0003 (95% CI, 1.00004 to 1.00068), *P* = .01	*P* = .22	NA
Prechemotherapy β-hCG: (IU/L)	OR, 0.99 (95% CI, 0.99 to 0.99), *P* = .01	*P* = .09	NA
Teratoma in initial pathology	OR, 2.75 (95% CI, 1.28 to 6.10), *P* = .01	*P* = .38	NA
Primary lesion being lymph node	OR, 3.13 (95% CI, 1.22 to 8.81), *P* = .02	*P* = .93	NA
Radiomics signature (R + miRNAs)	NA	***P* < .0001**	NA
Model performance	NA	***P* < .0001**	***P* = .02**

NOTE. Significant predictors identified in the univariate analysis were AFP levels (*P* = .01), β-hCG levels (*P* = .01), presence of teratoma in the primary (*P* = .01), and metastatic lymph node lesions (*P* = .02). The multivariate analysis incorporated these clinical features and the radiomics signature (radiomics features and miRNA expression levels). The radiomics signature was the strongest predictor with *P* values < .0001 and .02. Bolded values highlight the performance of the radiomics signature in the multivariate analysis, as well as the model performances in the training and test sets.

Abbreviations: AFP, alpha-fetoprotein; β-hCG, beta-human chorionic gonadotropin; NA, not applicable; OR, odds ratio.

## DISCUSSION

The most significant finding of this study is that ML models integrating radiomics features from CT images and miR371 and miR375 obtained before surgical resection can distinguish teratoma from nonteratoma histology in patients with mNSGCT presenting postchemotherapy residual disease (AUC of 0.96 in training and 0.83 in testing [95% CI, 0.68 to 0.98]). The proposed methodology can potentially improve treatment precision by reserving surgery for patients with residual teratoma or, in selected cases with vGCTs, sparing unnecessary surgery for patients with benign histology.

Predicting retroperitoneal histology of postchemotherapy disease in GCT has gained attention since the 90s and has mainly consisted of using clinical factors to predict residual disease histologies. Steyerberg et al^38^ developed a clinical model with the parameters post-treatment residual mass size, mass size change during chemotherapy, prechemotherapy tumor marker levels, and the teratoma histology in the primary that can help predict residual disease histology. Subsequently, Vergouwe et al pioneered the field's predictive model to distinguish between postchemotherapy necrosis/fibrosis and vGCT or teratoma with achieved AUC values ranging from 0.77 to 0.84.^39^ Clinical factors, however, have very limited value in identifying teratoma.^40^

Recent studies suggest that circulating miRNAs can help identify residual disease in testicular cancer. The miR371, expressed by vGCT, is inaccurate in detecting teratoma, presenting a gap in its clinical applications. Attempts to address this gap with miR375 have shown limited value. As described by Nappi et al,^30^ the combination of plasma miR371 and miR375 has an AUC of 0.77 in predicting residual teratoma, limiting its clinical utility.

In current clinical practice, only a small number of patients present residual active malignancy after chemotherapy.^41^ These patients are easily identified by either standard serum tumor markers or miR371, which is not detectable in teratoma.^37,42,43^ Thus, identifying teratoma is the primary unmet clinical need in managing postchemotherapy residual disease in mNSGCT. Building on this background, we hypothesize that integrating radiomics with miR371 and miR375 may improve the accuracy of detecting residual teratoma.

Model classifiers such as GB and SVM have been previously applied to testis cancer to evaluate radiomics prediction of malignant versus benign histologies. Baessler et al^22^ developed a CT radiomics–based ML classifier to predict the histopathology of lymph nodes (eg, benign—necrosis/fibrosis, or malignant–vGCT and/or teratoma) after postchemotherapy RPLN dissection. They showed that the GB model performed well in predicting malignancy (AUC: 0.81, SN: 88%, and SP: 72%). The SVM classifier had a discriminative accuracy of 72% for differentiating between vGCT/teratoma and fibrosis (AUC: 0.74, SN: 56.2%, SP of 81.9%).^44^ The model's discriminative accuracy improved from 72% to 88% when combined with clinical predictors.

CB has not been used to predict the histology of residual masses in testis cancer. In our study, CB integrating miR371 and miR375 demonstrated superior accuracy, with an AUC of 0.83 (95% CI, 0.68 to 0.98), SN of 0.71, and SP of 0.76. Moreover, the combination of CB radiomics and miRNAs independently predicted teratoma, while the clinical parameters did not demonstrate the ability to predict teratoma in the multivariate analysis.

Our observations were recently confirmed by Li et al. In this study, the combination of miR371, miR375, and radiomics achieved an AUC of 0.86 (95% CI, 0.72 to 1.0) for predicting teratoma histology in residual RPLNs. They also reported an AUC of 0.64 (95% CI, 0.41 to 0.87) for predicting vGCT and 0.78 (95% CI, 0.63 to 0.93) for predicting necrosis.^45^ Of note, this study was only conducted using RPLN residual lesions and the models were not constructed to detect teratoma specifically.

Overall, integrated CT radiomics and circulating miRNA modeling is a new strategy that has not been previously explored to specifically detect residual teratoma. The idea of combining features from different evaluation models results in a multidimensional assessment of the residual tissue, which has the potential to provide better predictive ability. Our study distinguishes itself from previous research that primarily focused on radiomic features in RPLNs. It incorporates diverse features (miRNAs and radiomic features) and lesion locations, reflecting routine clinical practice. Finally, our multivariate model, combining radiomics signature (including radiomics features and miRNAs) and clinical parameters, highlights the superior predictive power of the radiomics signature, indicating that imaging features captured by radiomics outperform those provided by clinical parameters. An important point is that, despite their univariate associations, clinical factors lost their significance in the multivariate analysis. The potential reasons for that could be radiomic features extracted from postchemotherapy imaging, which may encompass features from markers of tumor burden, histology, and some other relevant features, absorbing the contribution of clinical variables. Additionally, the temporal proximity of radiomics data may provide more accurate predictions than baseline clinical information.

The retrospective design and limited patient cohort may affect the generalizability of our findings. Despite improvements in prediction capability, the inconsistent results with various radiomics models from the literature indicate the need for improvement in modeling strategies and highlight the challenges these models face with different data sets and histologies.

Fully automated segmentation tools may also be critical for improving model robustness and generalizability since the current methodology is operator-dependent. Additionally, advanced ensemble models could leverage the strengths of different ML models to enhance overall discrimination power.^46,47^ Our findings underscore both the benefits and challenges associated with the integrated approaches incorporating both biomarker and radiomics, particularly highlighting the significant role of multiomics data integration with radiomic features in enhancing predictive accuracy. Although our study focuses on nongenomic data, integrating genomic features into the proposed models may offer detailed insights into the biological mechanism driving histological subtypes and their imaging correlates. Radiogenomics is an emerging field that combines radiomics and genomics features to overcome both approaches' shortcomings and enhance results. To date, there have been several reports on various cancers, including glioma, breast cancer, colorectal cancer, and clear cell carcinoma, reporting moderate to strong success with radiogenomics approaches detecting particular mutation or methylation patterns.^48,49^ Testicular cancer histological subtypes reveal genomic heterogeneities such as *KIT* and *KRAS* mutations in seminoma and chromosomal instability in teratomas, possibly influencing miRNA expression and imaging features in specific ways. Benign lesions may also reflect genomic scarring after treatment or epigenetic silencing.^50^ As such, linking radiomics features, miRNA expression patterns, and genomic features might better help with histology identification, which is an area for further investigation. However, it is also important to consider that overfitting remains a key concern when implementing multidimensional data.

Another caveat of almost all available data in the literature integrating biomarkers with radiomics is the possible limitations of building a classifier on the basis of multiple ROIs from the same patient with discordant lesions. Although the radiomics features are ROI-specific, miRNAs are lesion-specific and may pose limitations for data interpretation. In our cohort, there were no patients with discordant lesions (different lesion histology in the same patient). As such, this was not an issue affecting our analysis. However, multiple samples from the same patient still may compromise the data set's independence, introducing bias. With our study's relatively limited patient cohort, these factors may affect the generalizability of the findings. Therefore, collaboration is crucial to overcoming the limited sample size, possibly with independent patient samples. Despite all the limitations, promising findings reported here may provide a background for a follow-up prospective multicenter validation study. Moreover, in the era of an AI reproducibility crisis, where papers are published but cannot be reproduced, more elaborate descriptions of method specifics, radiomics features, ML details, and GitHub model sharing are needed.^51-53^ For the current paper, the complete code is publicly available at GitHub.^54^
